# Gene Expression Subtyping Reveals Immune alterations:TCGA Database for Prognosis in Ovarian Serous Cystadenocarcinoma

**DOI:** 10.3389/fmolb.2021.619027

**Published:** 2021-09-24

**Authors:** Chunxia Feng, Yan Xu, Yuanyuan Liu, Lixia Zhu, Le Wang, Xixi Cui, Jingjing Lu, Yan Zhang, Lina Zhou, Minbin Chen, Zhiqin Zhang, Ping Li

**Affiliations:** ^1^ Department of Radiotherapy and Oncology, The Second Affiliated Hospital of Soochow University, Suzhou, China; ^2^ Department of Radiotherapy and Oncology, Affiliated Kunshan Hospital of Jiangsu University, Kunshan, China; ^3^ Department of Oncology, The First Affiliated Hospital of Soochow University, Suzhou, China; ^4^ Clinical Research and Lab Center, Affiliated Kunshan Hospital of Jiangsu University, Kunshan, China; ^5^ Department of Gynecology, Affiliated Kunshan Hospital of Jiangsu University, Kunshan, China; ^6^ Department of Biobank, Affiliated Kunshan Hospital of Jiangsu University, Kunshan, China

**Keywords:** serous ovarian cancer, TCGA, tumor immune infiltration, prognosis, nomogram

## Abstract

Serous ovarian cancer is the most common and primary death type in ovarian cancer. In recent studies, tumor microenvironment and tumor immune infiltration significantly affect the prognosis of ovarian cancer. This study analyzed the four gene expression types of ovarian cancer in TCGA database to extract differentially expressed genes and verify the prognostic significance. Meanwhile, functional enrichment and protein interaction network analysis exposed that these genes were related to immune response and immune infiltration. Subsequently, we proved these prognostic genes in an independent data set from the GEO database. Finally, multivariate cox regression analysis revealed the prognostic significance of TAP1 and CXCL13. The genetic alteration and interaction network of these two genes were shown. Then, we established a nomogram model related to the two genes and clinical risk factors. This model performed well in Calibration plot and Decision Curve Analysis. In conclusion, we have obtained a list of genes related to the immune microenvironment with a better prognosis for serous ovarian cancer, and based on this, we have tried to establish a clinical prognosis model.

## Introduction

In female, ovarian cancer was the third most common cancer and the second leading cause of cancer-related deaths in 2018, as 295,414 people being diagnosed and 184,799 deaths being reported globally (Bray et al., 2018). Ovarian cancer is rarely detected early, as its symptoms are often not evident due to the anatomical location of the disease (Lheureux et al., 2019); therefore, several patients are diagnosed at an advanced cancer stage, which is associated with high mortality. Epithelial ovarian cancer accounts for 85–90% of all ovarian cancers and is the most common type of ovarian cancer with unique genomic characteristics such as mutations in BRCA1 and BRCA2 that encode proteins involved in DNA damaged repair. Hence, homologous recombination deficiency for DNA damage improves the precision and effectiveness of therapy (Rebbeck et al., 2015; Lheureux et al., 2019; Matsumoto et al., 2019). Serous histological subtype is the most common subtype of epithelial ovarian cancer, among which approximately 90% of cases are of high-grade serous ovarian cancer, whereas 10% are of low-grade cancers (Duska and Kohn, 2017). From a molecular and genetic point of view, serous is classified as a typical type I (low-grade) and type II (high-grade) ovarian cancer by the World Health Organization (WHO) (Previs et al., 2015), and its gene expression profile has molecular diversity. Overall, differences in clinical outcomes between molecular subtypes of ovarian cancer (Konecny et al., 2014; Winterhoff et al., 2016) allow the discovery of new predictors for individualized treatment.

Gene expression profiling has been widely used in ovarian cancer to identify distinct molecular subtypes. The Cancer Genome Atlas (TCGA) project provides gene expression data and clinical and survival information (Cancer Genome Atlas Resea, 2008). Ovarian serous cystadenocarcinoma (OV) is the most common histological subtype of epithelial ovarian cancer (Biade et al., 2006). In TCGA profile, four gene expression subtypes were independently identified within OV, including the immunoreactive, proliferative, mesenchymal, and differentiated subtype (Cancer Genome Atlas Resea, 2011). The ICON7 phase III trial revealed that the outcome of patients with ovarian cancer improved substantially regarding progression-free survival (PFS) when treated in combination with bevacizumab (Perren et al., 2011). Moreover, among OV patients treated with bevacizumab, those with proliferative and mesenchymal cancer subtypes had the most inferior survival, but derived a comparably more significant PFS benefit (hazard ratio [HR] 0.55 [95% confidence interval [CI] = 0.34–0.90], *p* = 0.016; and HR 0.78 [95%CI = 0.44–1.40], *p* = 0.41, respectively) (Kommoss et al., 2017). These two OV subtypes share an angiogenic gene expression signature and may respond to antiangiogenic therapy. Thus, molecular subtyping underscores the significant clinical need for more effective and refined treatment strategies (Kommoss et al., 2017).

Different gene expression profiles not only provide valuable clues regarding the molecular subtypes (Klymenko and Nephew, 2018; Ugo et al., 2018), but also the genetic pathways related to immune infiltration of tumor-associated normal cells (Sato et al., 2005; Mhawech-Fauceglia et al., 2013). ESTIMATE is an algorithm that infers tumor purity based on gene expression data of cancer samples comprising a mixture of stromal cells and immune cells (Yoshihara et al., 2013), which are essential components of normal cells in tumor tissues. These cells can interfere with molecular signals within the tumor microenvironment and are known to play crucial roles in cancer biology (Yoshihara et al., 2013) , with increasing evidence demonstrating the clinical importance of stromal and immune cells in tumor microenvironment (Liu et al., 2018; Xu et al., 2019; Mao et al., 2020). The immune microenvironment is involved in tumorigenesis and homeostasis in body, with tumor-infiltrating lymphocytes and stromal cells being associated with clinical prognosis in ovarian cancer patients (Zhang et al., 2003; Sato et al., 2005). However, only few studies focusing on whether the differences between the molecular subtypes of ovarian cancer are related to the tumor microenvironment.

The role of tumor microenvironment in predicting clinical outcome and efficacy was gradually recognized. Researchers were trying to find reliable immune-related prognostic genes in ovarian cancer, including TAP1 (Liu et al., 2020; Wu et al., 2020; Huo et al., 2021) and CXCL13 (Liu et al., 2020; Wu et al., 2020; Li et al., 2021). The results were based on screening for differentially expressed genes between high and low abundance immune subtypes (Li et al., 2021), ovarian cancer and normal tissues (Liu et al., 2020), high and low Tumor Mutational Burden (TMB) samples (Huo et al., 2021), high and low tumor microenvironment scores (Wu et al., 2020). However, TAP1 and CXCL13 as main prognostic markers were primarily carried out using a univariate Cox model (Wu et al., 2020), without considering the influence of other genes. In addition, some study included more genes in the prognostic model, which may increase the difficulty of application (Liu et al., 2020).

This study aims to screen the immune-related genes of serous ovarian cancer from different perspective based on the differences in the prognosis and immune scores among four molecular types. Moreover, we establish a new risk classification system based on two immune-related prognostic genes to predict overall survival, and propose potential prognostic markers and therapeutic targets for advanced ovarian cancers.

## Materials and Methods

### Patient Samples

Ovarian serous cystadenocarcinoma gene expression data by AffyU133a array was obtained from TCGA dataset (https://tcga-data.nci.nih.gov/tcga/) on June 7, 2020. In TCGA data portal, we also downloaded gene expression subtype (*n* = 308) and clinical data such as age, pathological stage, grade, and survival information. The level of stromal cells and the infiltration level of immune cells in OV tissues were viewed by scores from the ESTIMATE website (https://bioinformatics.mdanderson.org/estimate/index.html). The gene expression subgroups were used as the test group, while all TCGA data of OV were used as the internal verification group for the survival prognosis of the selected genes. GSE32062 data set(27) from the GPL6480 platform was downloaded for external verification.

### Differentially Expressed Genes

Package limma (Ritchie et al., 2015) was performed in data analysis to compare the differential expressed genes. Fold change (FC) > 1.5 and *p*-value < 0.05 were set as the cutoff for screening significant DEGs. Volcanic maps (R package) and Heatmaps were used to visualize the DEGs. The heatmaps and clustering were based on an open-source web tool Morpheus (https://software.broadinstitute.org/morpheus).

### Oncomine Analysis

Oncomine (https://www.oncomine.org/) is a database for tumor-related gene research from GEO, TCGA, and published literature. The transcription levels of up-regulated genes with prognostic significance were compared with those of normal control samples. The statistical method used for comparison was Student’s *t*-test.

### GEPIA Dataset

This tool was developed by Tang et al. (2017), for analyzing the RNA sequencing expression data from TCGA and the GTEx projects. The expression of up-regulated genes with prognostic significance in tumor tissues and control ones was carried out by this dataset (http://gepia.cancer-pku.cn/).

### Metascape Analysis

Metascape is a web-based portal (http://metascape.org), which provided comprehensive gene list annotation and analysis resources. Gene Ontology (GO) process and Kyoto Encyclopedia of Genes and Genomes (KEGG) pathways were conducted in functional enrichment analysis. Only terms with *p*-value < 0.01, minimum overlap of 3, and enrichment factor of >1.5 were considered significant. To understand the underlying interaction, Metascape was also employed to construct protein network analysis. Molecular Complex Detection (MCODE) algorithm was applied to identify densely connected network components. The results of enrichment analysis with prognostic genes were shown by a bubble diagram using an online platform (http://www.bioinformatics.com.cn).

### Overall Survival Curve

The prognostic significance of differentially expressed genes was assessed by univariate analysis. *p*-value < 0.05 was set as a threshold. Genes with predictive value were verified and visualized by Prism 7. A forest plot was used to visualize the verification survival results. These statistical analyses were performed using R (“survival” package, “forest plot” package).

### TIMER Analysis

Differentially expressed genes may play crucial roles in immune infiltration of tumor microenvironment. TIMER (Li et al., 2017a) was used to analyze the immune infiltration of DEGs, and gene modules of TIMER were performed to explore immune infiltrates through B cells, CD4^+^ T cells, CD8^+^ T cells, neutrophils, macrophages, and dendritic cells (Summerfield et al., 2001).

### Tissue Samples

Fresh ovarian cancer tissues and adjacent normal ovarian tissues were obtained from the Biobank of Affiliated Kunshan Hospital of Jiangsu University. These samples were obtained recently from 14 high-grade serous ovarian cancer patients, including two in stage I, four in stage II, five in stage III, and three in stage IV. Written informed consent was obtained from each patient and the protocols were approved by the Ethics Committee of Affiliated Kunshan Hospital of Jiangsu University (BR2015021).

### Quantitative Real-Time Reverse Transcriptase Polymerase Chain Reaction Assay

The detailed protocols were well established and described in our previous studies (Wang et al., 2020). In brief, TRIzol reagents were added to tissue specimens to obtain total RNA, which was reversely transcripted to cDNA. Through an ABI Prism 7900 Fast Real-Time PCR System, qRT-PCR was performed through the SYBR Green PCR kit. Melt curve analysis was always performed to calculate the product melting temperature, and the 2-ΔΔCt method was utilized to quantify targeted mRNA, with β-actin mRNA examined as the internal control. All the mRNA primers were provided by GENEWIZ (Suzhou, China) and the sequences of the primer were listed in Table1. The experiment was repeated 3 times. The unpaired *t*-test was used to compare the difference between the tumor tissues and normal ones.

**TABLE 1:** Primers used in the study.

qPCR primers	
*β-actin* Forward	5′-TCA​AGA​TCA​TTG​CTC​CTC​CTG​AG- 3′
*β-actin* Reverse	5′-ACA​TCT​GCT​GGA​AGG​TGG​ACA- 3′
*TAP1* Forward	5′-CTG​GGG​AAG​TCA​CCC​TAC​C- 3′
*TAP1* Reverse	5′-CAG​AGG​CTC​CCG​AGT​TTG​TG- 3′
*CXCL13* Forward	5′-GCT​TGA​GGT​GTA​GAT​GTG​TCC- 3′
*CXCL13* Reverse	5′-CCC​ACG​GGG​CAA​GAT​TTG​AA- 3′

### cBioPortal Database

The cBioPortal website (https://www.cbioportal.org/) stores DNA copy number data, mRNA and microRNA expression data, and other useful information. Samples with mRNA data (RNA Seq V2) of 585 ovarian cancer data were obtained from the cBioPortal to further analyze the genetic alterations and Co-expression of TAP1 and CXCL13. The first 100 co-expressed genes of CXCL13 and TAP1 were obtained from the cBioPortal database. The protein-protein interaction network of shared genes was retrieved from the STRING database (high confident: 0.7) (https://string-db.org/). The network was further constructed by Cytoscape software.

### Nomogram Model

Cox proportional hazards regression model was used for multivariate analysis of prognostic factors. The heatmap of the relationship between risk classification and gene expression was drawn on the sangerbox website (http://sangerbox.com/). The regression coefficients in the multivariate Cox regression model were used to generate a nomogram. The performance of the nomogram was evaluated using the Calibration plot, C index, and Decision Curve Analysis. All statistical analyses were performed using R (R version3.5.1; Institute for Statistics and Mathematics, Vienna, Austria). “rms” package, “survival” package, “survivalROC” package and “pheatmap” package were installed and used.

## Results

### Gene Expression Subtypes Are Significantly Associated With Immune Score and Prognosis

Gene expression array and clinical information of all 593 samples were downloaded from TCGA database, including 568 primary OV cases, 17 recurrent tumor tissues, and eight normal tissue samples. The enrolled patients were initially diagnosed between 1992 and 2013 and were followed up from 2009 to 2015. The array contained 308 OV cases with specific gene expression subtypes, including 84 (27.27%) cases of immunoreactive subtype, 85 (27.60%) cases of proliferative subtype, 69 (22.40%) cases of mesenchymal subtype, and 70 (22.73%) cases of differentiated subtype. Gene expression types were not statistically significant with pathological stage (*p* = 0.092), grade (*p* = 0.861), new event type in the course of the disease (*p* = 0.141), cancer status at enrollment (*p* = 0.335), anatomic site (*p* = 0.577), and initial pathologic diagnosis method (*p* = 0.402), but were related to age (*p* = 0.002) and OS (*p* = 0.035) (Table 2). It has been shown that clinical characteristics including the initial pathologic diagnosis method have little effect on gene expression subtypes.

**TABLE 2:** TCGA baseline clinical characteristics of gene expression subtypes and single-factor analysis.

Gene_expression_subtypes						
Characteristics	Total	Differentiated	Immunoreactive	Mesenchymal	Proliferative	*p* value
Age	59.12 ± 10.977	55.14 ± 9.38	58.69 ± 10.99	61.23 ± 11.30	61.06 ± 11.16	0.002*
OS time	949 (442–1579)	1032 (449.5–1488)	1059 (448–1754.5)	787 (481.5–1366.5)	907.5 (369.25–1685.75)	0.574
OS						
Live	120	22	44	23	31	0.035*
Dead	183	47	37	46	53	
no available	5	1	3	0	1	
Stage						
I	1	0	0	1	0	0.092
II	21	5	8	1	7	
III	242	48	65	60	69	
IV	38	16	8	6	8	
no available	6	1	3	1	1	
Grade						
G1	1	0	0	1	0	0.861
G2	35	7	7	11	10	
G3	261	61	73	56	21	
G4	1	0	0	0	1	
GX	4	1	1	1	1	
no available	6	1	3	0	2	
New event type						
locoregional Disease	4	3	1	0	0	0.141
metastatic	1	0	0	1	0	
progression of Disease	12	2	2	1	7	
recurrence	151	33	39	37	42	
no available	140	32	42	30	36	
Cancer status						
tumor free	69	16	20	13	20	0.335
with tumor	197	49	47	47	54	
no available	42	5	17	9	11	
Anatomic site						
Bilateral	209	52	51	48	58	0.577
Left	36	6	13	7	10	
Right	42	11	12	8	11	
no available	21	1	8	6	6	
Initial pathologic diagnosis method						
cytology (e.g., peritoneal or pleural fluid)	27	5	9	10	3	0.402
excisional Biopsy	5	2	1	2	0	
fine needle aspiration biopsy	3	0	1	1	1	
tumor resection	265	61	70	56	78	
other method	8	2	3	0	3	

*Statistically significant (*p*-value < 0.05).

The four gene expression subtypes of OV are differentiated, immunoreactive, mesenchymal, and proliferative types in TCGA.

**FIGURE 1:**
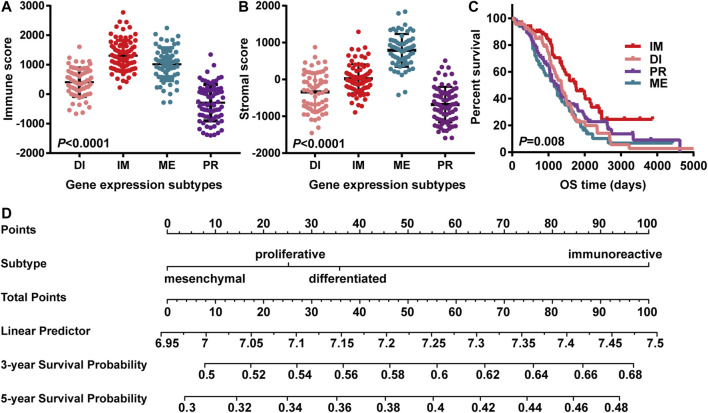
OV gene expression subtypes were significantly associated with immune score and overall survival. “IM” “PR” “ME” “DI” indicated “immunoreactive type” “proliferative type” “mesenchymal type” and “differentiation type,” respectively. **(A)** Distribution of immune scores; OV subtypes had significant correlations with immune scores; represented by the histogram (*n* = 308, *p*-value < 0.05); **(B)** Distribution of stromal scores; OV subtypes had significant correlations with stromal scores; represented by the histogram (*n* = 308, *p*-value < 0.05); **(C)** The survival relationship between the immunoreactive group and the other groups. Kaplan Meier’s survival curve showed that the prognosis of the immunoreactive group was better than that of the other groups, as demonstrated by log-rank, *p*-value < 0.05. **(D)** The nomogram showed the 3 years and 5 years survival probability among different subgroups. The immunoreactive subgroup scored higher than the rest ones.

ESTIMATE was used to compare the infiltration of immune cells of the four types. Based on the ESTIMATE algorithm, stromal scores ranged from -1593.24 to 1837.43, and immune scores were distributed between −1400.6 and 2774.16. Among the immune scores of ovarian cancers, the average score of immunoreactive type was the highest, followed by mesenchymal and differentiation ones, and proliferative type had the lowest score (Figure 1A). On the stromal score, the mesenchymal type got the highest score above the immunoreactive type, and the lowest score belonged to the proliferative type (Figure 1B).

The overall survival was further analyzed in the four gene expression subgroups with the available immune scores. Gene expression matrix and clinical information of 308 cases were extracted to analyze the influence of different gene subtypes on prognosis. Compared with mesenchymal type (HR = 0.52, 95%CI 0.33 to 0.82, *p*-value = 0.00), differentiation type (HR = 0.59, 95%CI 0.38 to 0.91, *p*-value = 0.01), and proliferative type (HR = 0.62, 95%CI 0.40 to 0.95, *p*-value = 0.03), Kaplan-Meier survival curves showed that the immunoreactive type had the best prognosis (*p*-value = 0.008) (Figure 1C). Subsequently, we combined the gene subtypes, immune scores, and stromal scores to further demonstrate the impact on 3 years and 5 years survival of ovarian cancer through the nomogram. This figure also suggests that immunoreactive type patients have a better survival probability (Figure 1D).

### Comparison of Differentially Expressed Genes Among Gene Expression Subtypes

**FIGURE 2:**
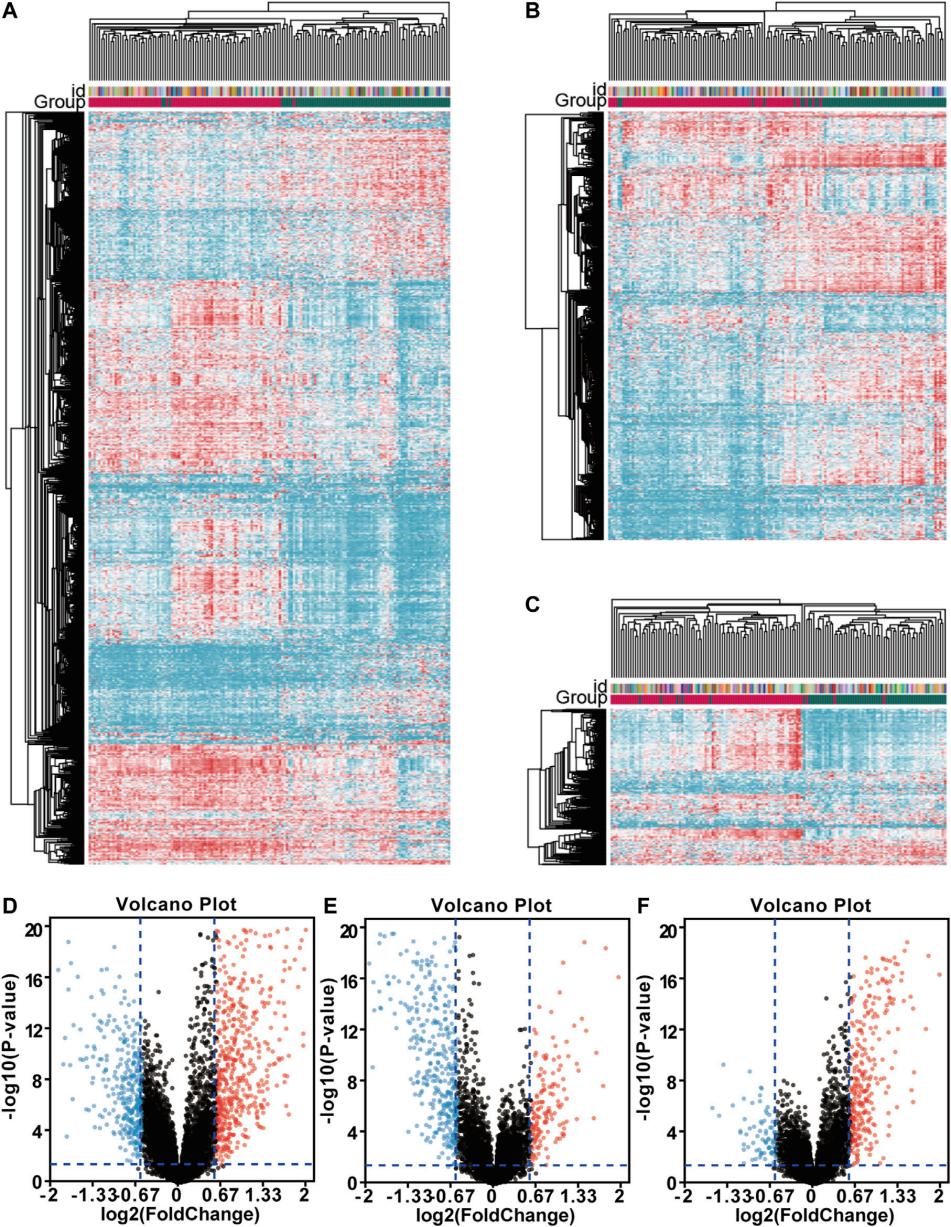
Comparison of differentially expressed genes (DEGs) among gene expression subtypes. The drawing of the heatmap was based on the Hierarchical clustering and Euclidean distance measurement method. The volcano map was drawn using online tools (http://sangerbox.com/). Red and blue indicated upregulated and down-regulated differential genes. Fold change (FC) > 1.5 and *p*-value < 0.05; **(A)** Heatmap of DEGs between the immunoreactive group and proliferative group; **(B)** Heatmap of DEGs between the immunoreactive group and mesenchymal group; **(C)** Heatmap of DEGs between the immunoreactive group and differentiation type; **(D)** The volcano map between the immunoreactive group and proliferative group; **(E)** The volcano map between the immunoreactive group and mesenchymal group; **(F)** The volcano map between the immunoreactive group and differentiation type.

In this section, we identified the DEGs between the immunoreactive subtype and other subtypes to reveal the correlation of survival differences. First, the difference between gene expression subtypes was analyzed by package limma. Then, we applied heatmaps to show the differential gene expression matrix. Hierarchical clustering was used to cluster different subtypes, and we measured them by the Euclidean distance method. Compared with the proliferative group, 607 genes were upregulated, and 356 genes were down-regulated in the immunoreactive group. 138 genes were upregulated, and 412 genes were down-regulated when compared with the mesenchymal group; 276 genes were upregulated, and 72 genes were down-regulated when compared with differentiation type. Upregulated and downregulated genes in each comparison group were visualized using heatmaps (Figures 2A–C) and volcanic maps (Figures 2D–F).

### Functional Enrichment Analysis and Protein-Protein Interactions Among Differentially Expressed Genes Between Subtypes

**FIGURE 3:**
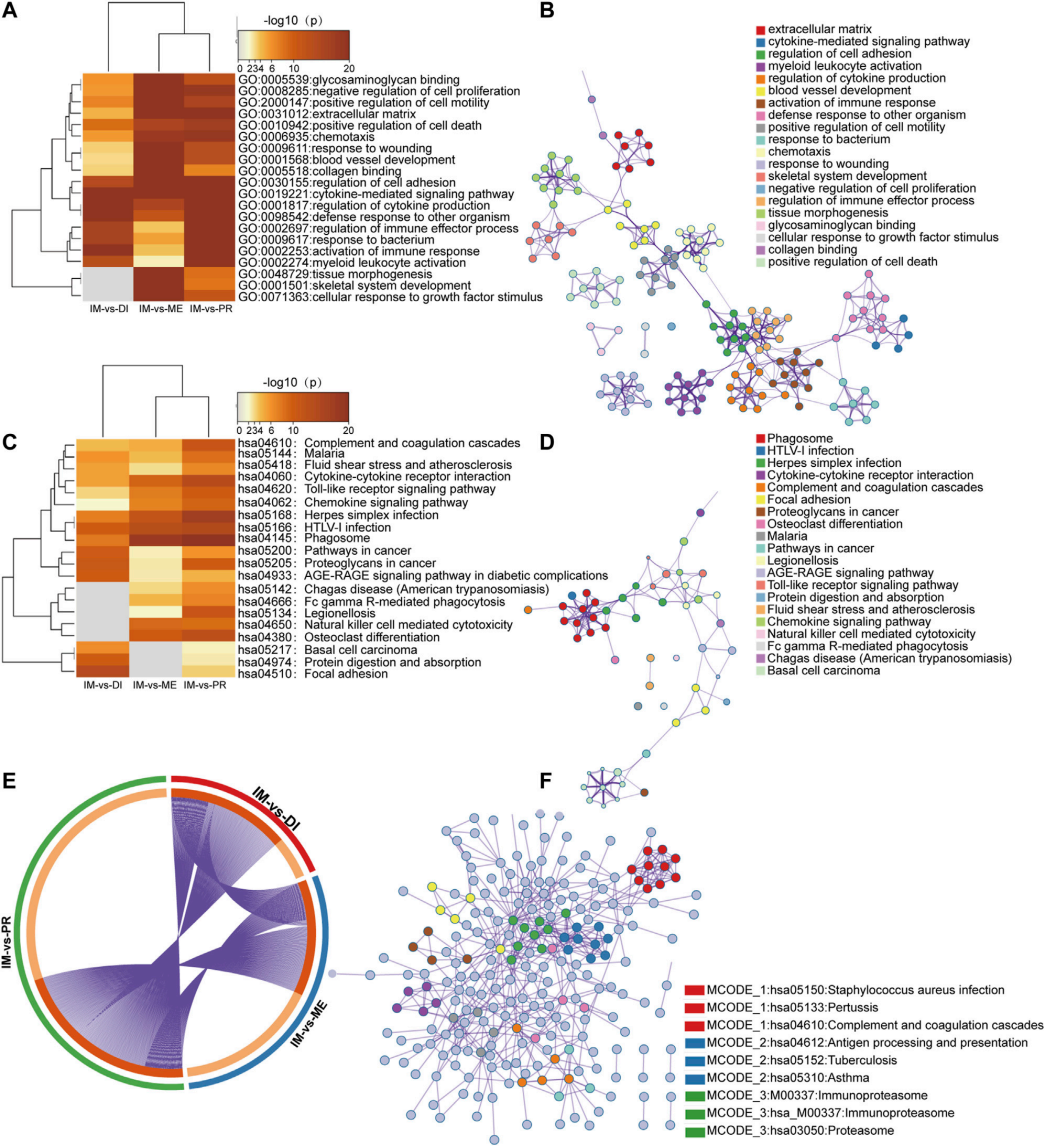
Functional enrichment analysis and protein-protein interactions across three lists of DEGs between subtypes. Heatmap of GO terms **(A)** and KEGG pathway **(C) **enriched across three DEGs lists. A network of enriched terms was colored by cluster-ID, where nodes shared the same cluster-ID were typically close to each other, **(B)** GO enrichment terms; **(D)** KEGG pathway enrichment terms. **(E)** The overlap between three DEGs lists. The outer circle showed each comparison group, and the inner circle represented the distribution of DEGs in each group. Genes that hit the multigroup list were shown in dark orange and those genes that did not were shown in light orange. **(F)** Protein-protein interactions network and MCODE components of the KEGG pathway. The top three elements of MCODE were expressed in detail.

To analyze the potential function of DEGs between the immunoreactive group and other groups, we performed functional enrichment analysis and protein-protein interactions. Gene ontology (GO) terms showed the analysis results through Biological Processes, Cellular Components, and Molecular Functions. In the immunoreactive vs. proliferative groups and immunoreactive vs. differentiated groups, functional concentration mainly focused on immune response, immune regulation, and defense response. While immunoreactive vs. mesenchymal subtypes primarily concentrated on the regulation of chemotaxis and cytokine and the formation of extracellular matrix, blood vessels, and other tissue structures (Figures 3A,B,E).

KEGG analysis concentrated on infection, cancer, chemokine signaling pathway, cytokine action, phagocytosis, and the inflammatory response when compared immunoreactive to proliferative types. Immunoreactive vs. differentiated groups were focused on infection, cancer, and inflammatory response. Immunoreactive vs. mesenchymal groups were concentrated in infection, cytokines, and inflammation (Figures 3C,D,E).

Metascap uses BioGrid, InWeb_IM, OmniPath databases to analyze interactions between proteins. Enrichment analysis is a collection of physically related proteins. If the network contains 3 and 500 proteins, the MCODE algorithm is used to identify the network components. Figure 3F showed the protein-protein interaction network analyzed by KEGG pathway enrichment analysis, and it expressed the first three components of MCODE in detail.

### Prognostic Value of Differentially Expressed Genes in Overall Survival

**FIGURE 4:**
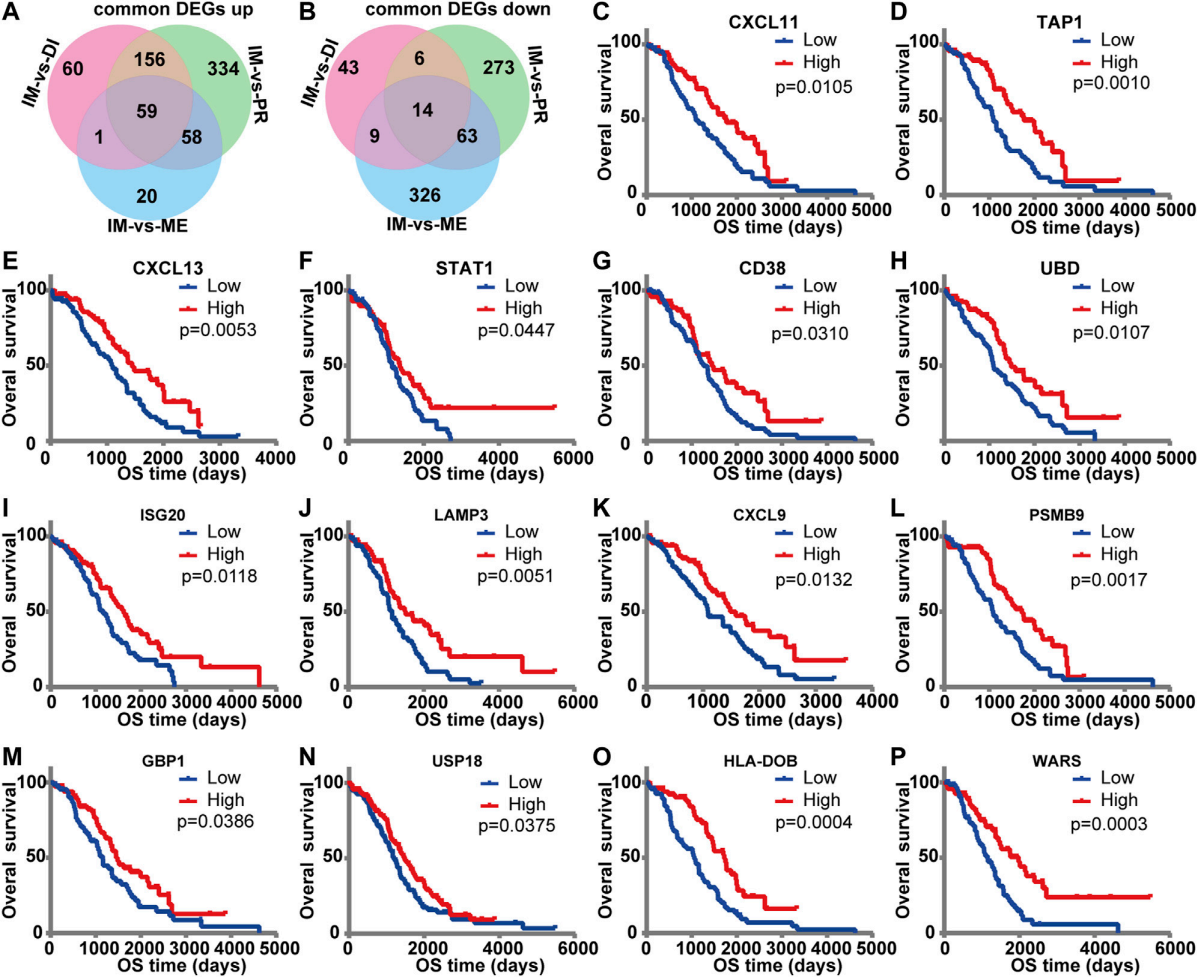
The prognostic value of DEGs for overall survival in the TCGA database. Venn diagram of common DEGs. It showed the common upregulated **(A)** and down-regulated **(B)** differential genes in the three comparison groups. **(C–P)** Kaplan‐Meier survival curves were used to identify common differential genes with prognostic significance. The influence of high and low expression of each DEG on overall survival was compared. The red line indicated high expression, and the blue line indicated low expression. *p* < 0.05 was considered as prognostic.

Venn diagram was used to explore the common DEGs from three comparison groups. It was found that 59 common genes were upregulated, and 14 common genes were down-regulated (Figures 4A,B). To further investigate the relationship between these common DEGs and prognosis, we evaluated each common gene by univariate survival analysis. Among the 59 upregulated genes, 14 genes were associated with better prognosis of ovarian cancer (HR < 1, *p* < 0.05), including CXCL11, TAP1, CXCL13, STAT1, CD38, UBD, ISG20, LAMP3, CXCL9, PSMB9, GBP1, USP18, HLA-DOB, WARS (Figures 4C–P).

**FIGURE 5:**
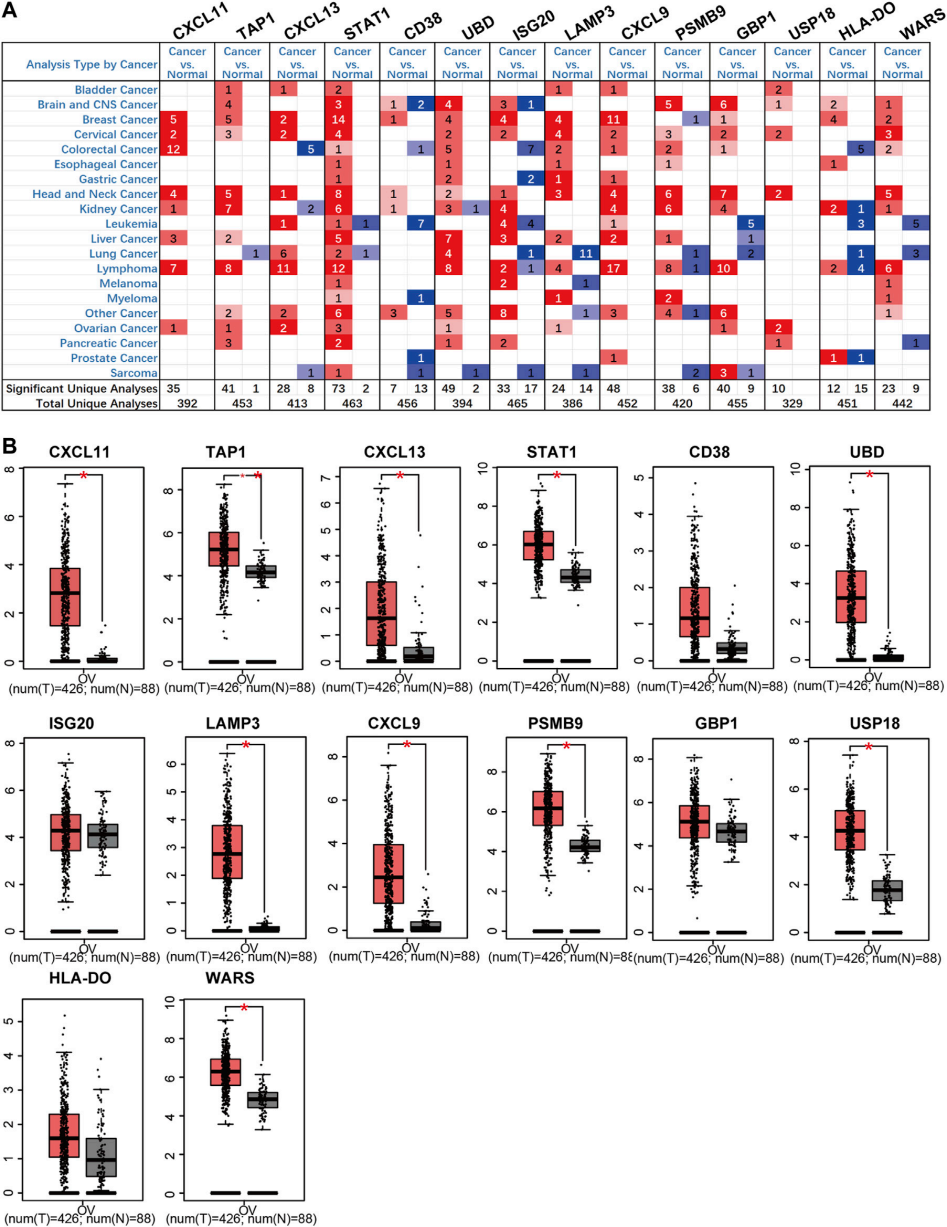
The mRNA expression of up-regulation prognostic genes in ovarian cancer tissues and normal tissues (Oncomine and GEPIA). **(A)** The mRNA expression on the Oncomine. **(B)** The mRNA expression on the GEPIA.

According to the Oncomine and GEPIA databases, mRNA expression of these screened genes was compared between ovarian cancer and normal ovarian tissues. The Oncomine database revealed that expression levels of CXCL11, TAP1, CXCL13, STAT1, UBD, LAMP3, GBP1, and USP18 increased in ovarian cancer. Also, the transcriptional levels of CD38, ISG20, CXCL9, PSMB9, HLA-DO, and WARS had no clear statistical results (Figure 5A). While in the GEPIA database, the results indicated that the expression of these genes in tumors was higher than that of normal controls, and the expression of CXCL11, TAP1, CXCL13, STAT1, UBD, LAMP3, CXCL9, PSMB9, USP18, and WARS was increased significantly in ovarian cancer samples (Figure 5B).

### Functional Enrichment Analysis and Protein-Protein Interactions Among Genes With Prognostic Value

**FIGURE 6:**
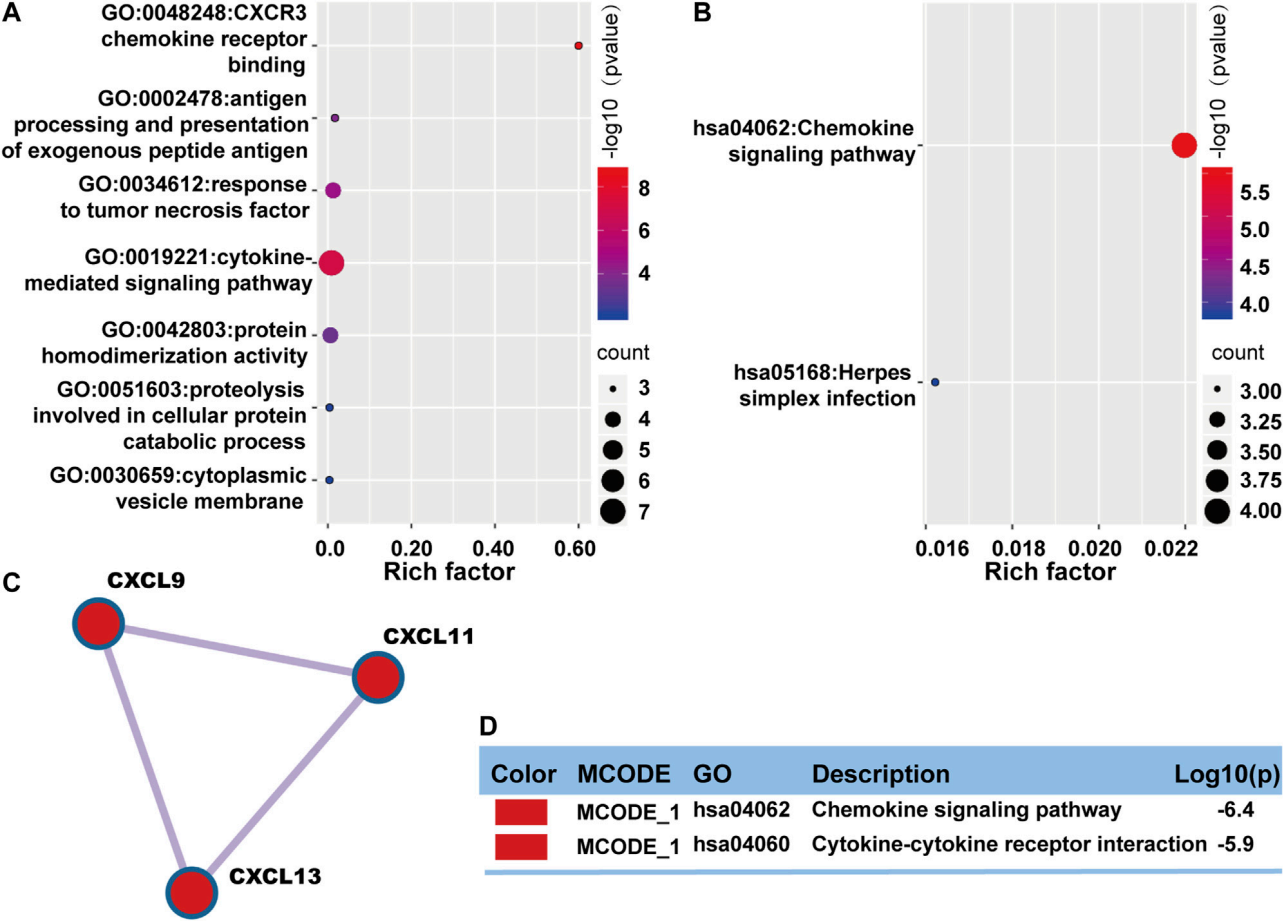
Functional enrichment of common DEGs with prognostic value in OV. **(A)** A bubble plot displaying GO enrichment analysis. The enriched terms were colored by *p*-value, which contained more genes that leaned towards having a larger bubble. **(B)** A bubble plot describing KEGG enrichment analysis. The enriched terms were colored by *p*-value, which contained more genes that leaned towards having a larger bubble. **(C)** One MCODE was formed in the protein-protein interaction network. **(D)** The MCODE was described by functional enrichment analysis.

Enrichment analysis and protein-protein interactions network were performed on Metascape again to understand the interrelationship between these prognostic genes better. Gene ontology enrichment analysis mainly focused on immune response, inflammatory response, and protein metabolism (Figure 6A). KEGG pathway was mostly focused on immune response and infection (Figure 6B). Protein-protein interactions network analysis showed only one MCODE component involved three protein components: CXCL9, CXCL11, and CXCL13 (Figure 6C). The main functions were focused on chemokine signaling pathway and cytokine-cytokine receptor interaction (Figure 6D).

### Validation in The Cancer Genome Atlas and GEO Databases

**FIGURE 7:**
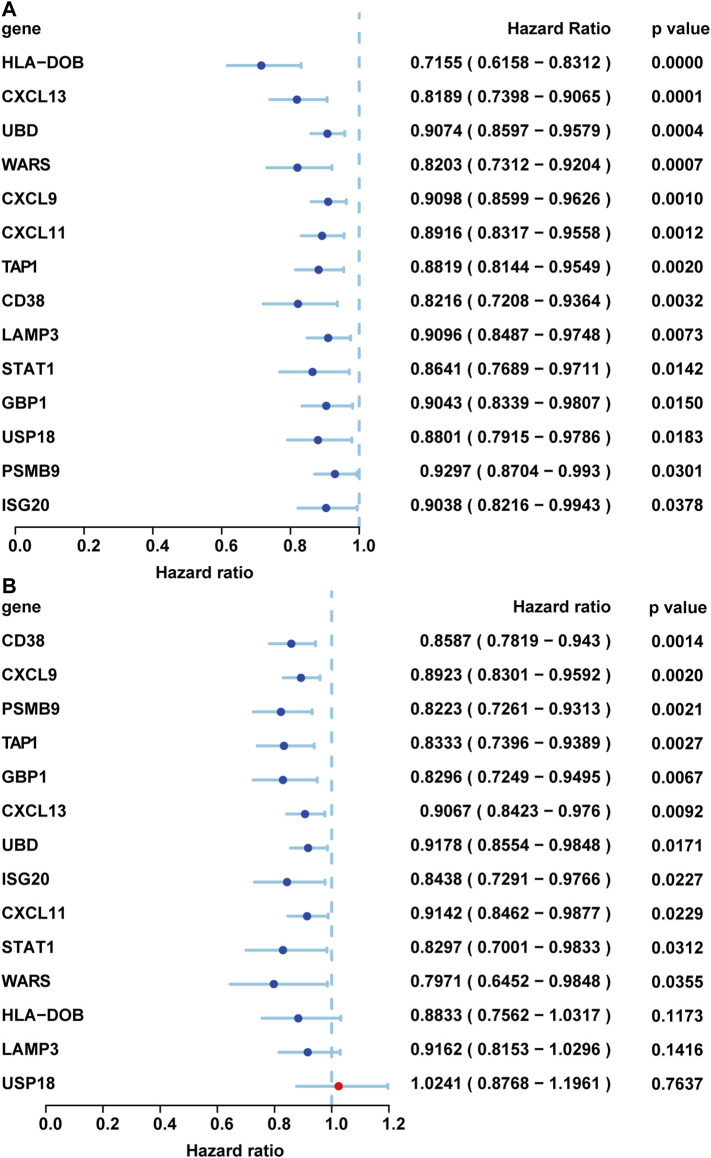
Validation in the TCGA and GEO databases. The prognostic verification results of each gene were displayed through the forest plot. **(A)** Internal verification by TCGA database. **(B)** External validation by GSE 32062 profile. These genes were sorted by *p*-value.

Then the survival significance of these genes was verified in whole TCGA Ovarian Cancer database and another GSE32062 database containing serous ovarian cancers (Yoshihara et al., 2012; Fan et al., 2020a; Zhao et al., 2019). We first chose internal validation. We analyzed the expression data and OS of 568 primary OV cases in TCGA database. The samples with no survival information and the survival time within 30 days were both removed. Finally, 548 primary OV samples were included in the statistical analysis (Supplementary Table S1). We found that all 14 genes with prognosis were verified with a better prognosis (HR < 1, *p*-value < 0.05) (Figure 7A). To further confirm whether these genes have prognostic significance, the GSE32062 data set was used for external verification (Supplementary Table S1). The gene expression in the two datasets showed a significant positive correlation (Zhao and Fan, 2019). We extracted serous ovarian cancers in this data set and found that 11 of the upregulated genes had better prognostic significance, including CD38, CXCL9, PSMB9, TAP1, GBP1, CXCL13, UBD, ISG20, CXCL11, STAT1, and WARS (Figure 7B).

### Correlation Between Verified Prognosis-Related Differentially Expressed Genes and Immune Cell Infiltration

Functional enrichment of these common differential genes with prognostic significance was conducted. We found that the focus was mainly on chemokine, cytokine, antigen processing and presentation, infection related to inflammatory responses, and immune responses. Therefore, we further analyzed the immune cell infiltration mediated by these genes. The scatterplots were generated and displayed on the TIMER website, as the purity-corrected partial Spearman’s rho value and statistical significance were showed. The results showed that DEGs were negatively correlated with purity, and these validated prognostic genes were all positively correlated with six kinds of immune-related cells: B cell, CD8^+^ T cell, CD4^+^ T cell, macrophage, neutrophil, and dendritic cell, though ISG20 and UBD were negatively correlated with macrophage infiltration (*p*-value > 0.05). Among them, six immune cells infiltration were significantly associated with the expression of STAT1, CXCL11, CXCL13, PSMB9, and GBP1, but no macrophage cell infiltration was found statistically significant (*p*-value < 0.05) for the remaining six genes: CD38, CXCL9, TAP1, WARS, ISG20, and UBD (Table 3).

**TABLE 3:** The correlation between prognosis-related DEGs and immune cell infiltration.

Gene	Purity		B Cell		CD8+ T Cell		CD4+ T Cell		Macrophage		Neutrophil		Dendritic Cell	
	partial.cor	*p*	partial.cor	*p*	partial.cor	*p*	partial.cor	*p*	partial.cor	*p*	partial.cor	*p*	partial.cor	*p*
CXCL11	−0.35	*	0.27	*	0.36	*	0.22	*	0.12	*	0.47	*	0.40	*
TAP1	−0.30	*	0.27	*	0.34	*	0.20	*	0.00	>0.05	0.43	*	0.38	*
PSMB9	−0.31	*	0.33	*	0.44	*	0.19	*	0.12	*	0.50	*	0.46	*
CXCL9	−0.48	*	0.20	*	0.40	*	0.27	*	0.03	>0.05	0.34	*	0.39	*
UBD	−0.38	*	0.21	*	0.30	*	0.24	*	−0.05	>0.05	0.36	*	0.36	*
CD38	−0.36	*	0.21	*	0.37	*	0.28	*	0.09	>0.05	0.42	*	0.40	*
GBP1	−0.35	*	0.25	*	0.34	*	0.19	*	0.02	*	0.44	*	0.37	*
CXCL13	−0.43	*	0.11	*	0.34	*	0.31	*	0.06	*	0.37	*	0.36	*
WARS	−0.29	*	0.16	*	0.23	*	0.19	*	0.05	>0.05	0.37	*	0.38	*
ISG20	−0.17	*	0.05	>0.05	0.14	*	0.15	*	−0.01	>0.05	0.26	*	0.16	*
STAT1	−0.21	*	0.19	*	0.25	*	0.12	*	0.16	*	0.33	*	0.25	*

*Statistically significant *(p-*value < 0.05).

### Establishment of Risk Score Formula

**FIGURE 8:**
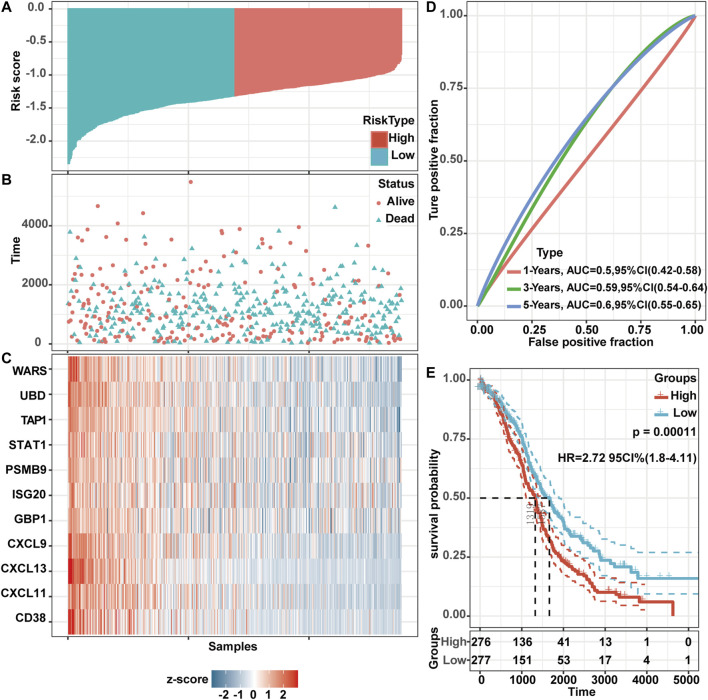
TAP1 and CXCL13 were significantly related to the OS of OV patients through multivariate cox regression analysis in TCGA. Risk score distribution **(A)**, survival status **(B)**, and validation DEGs expression **(C)** for multivariate analysis for patients in high-risk and low-risk groups. **(D)** Time-dependent ROC curve analysis. AUCs were set at 1 year, 3 years, and 5 years to evaluate the accuracy of the prognosis. **(E)** Kaplan-Meier curve analysis of OS in the low- and high-risk groups. The log-rank test was used to calculate the *p*-value.

To further evaluate the prognostic value of these screened genes, we used Cox proportional-hazards model to evaluate the impact of these gene expression on the prognosis. This analysis yielded a risk score. Based on this score, the OV samples in TCGA were divided into two parts: the high-risk group and the low-risk group (Figure 8A). The dot plot showed the survival status of these patients (Figure 8B). The following heatmap also showed the expression levels of these genes at different risk levels (Figure 8C). For high-risk patients, the expression level of these genes was decreased, while the expression level in the low-risk group was generally higher. Time-dependent ROC analysis was used to assess the predictive ability of these genes. The AUC was 0.50 (95% CI 0.42–0.58), 0.59 (95% CI 0.54–0.64), and 0.60 (95% CI 0.55–0.65) for 1 year, 3 years, and 5 years (Figure 8D). Kaplan Meier survival analysis described a poor prognosis for patients at high-risk status (log-rank test, HR 2.72, 95% CI 1.80–4.11, *p*-value = 0.00011) (Figure 8E), while TAP1 and CXCL13 were statistically significant in multivariate cox regression analysis (TAP1: *p*-value = 0.04520; CXCL13: *p*-value = 0.00287). Further, we set up a risk score formula based on the two genes, risk score = TAP1*(−1.2281) + CXCL13 *(−0.8237).

### Genetic Alterations and Co-expression of TAP1 and CXCL13

**FIGURE 9:**
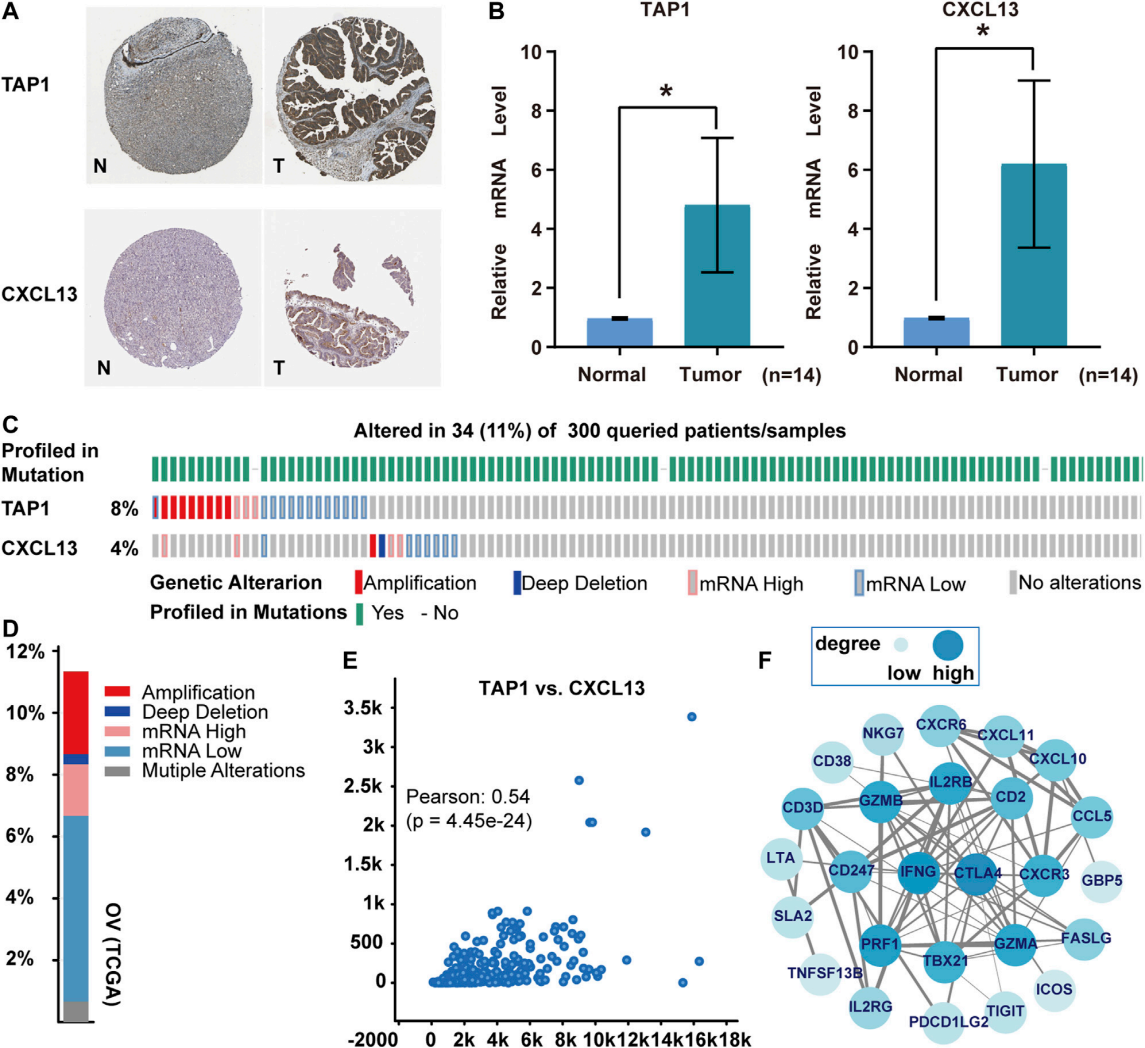
Protein expression and mutation analysis of TAP1 and CXCL13 in ovarian cancer. **(A)** Protein expression on the Human Protein Atlas. “N” was normal tissue, “T” was tumor tissue. **(B)** *TAP1 mRNA* and *CXCL13 mRNA* by qRT-PCR in ovarian cancer tissues and surrounding normal tissues (*n* = 14). *p < 0.05. **(C, D)** Genetic alterations on the cBioPortal. **(E)** Correlation between TAP1 and CXCL13. **(F)** Network of most frequently altered neighbor genes (combined score >0.7). The thickness of the connecting line indicated the level of combined-score, the higher the score, the thicker the line.

TAP1 and CXCL13 were risk factors that affect the prognosis of ovarian cancer. The high protein expression of TAP1 and CXCL13 in ovarian cancer was observed in the Human Protein Atlas (HPA) database (https://www.proteinatlas.org/) (Figure 9A). Meanwhile, *TAP1 mRNA* and *CXCL13 mRNA* levels were significantly elevated in ovarian cancer tissues compared to normal ones (Figure 9B). On the other hand, the genetic alterations, correlations, and co-expressed genes of TAP1 and CXCL13 were calculated using the cBioPortal. It showed that the alteration rate of these two genes in 300 mRNA data was 11%, including amplification, deep deletion, mRNA high, and mRNA low (Figures 9C,D). The Pearson correlation was 0.54 in positive correlation (Figure 9E). Subsequently, the top 100 genes related to TAP1 and CXCL13 were intersected to construct a network for frequently altered neighbor genes. CTLA4, IFNG, and PRF1 had higher degree values (Figure 9F).

### Construction and Verification of a Prognostic Risk Model for Advanced Ovarian Cancer Patients

**FIGURE 10:**
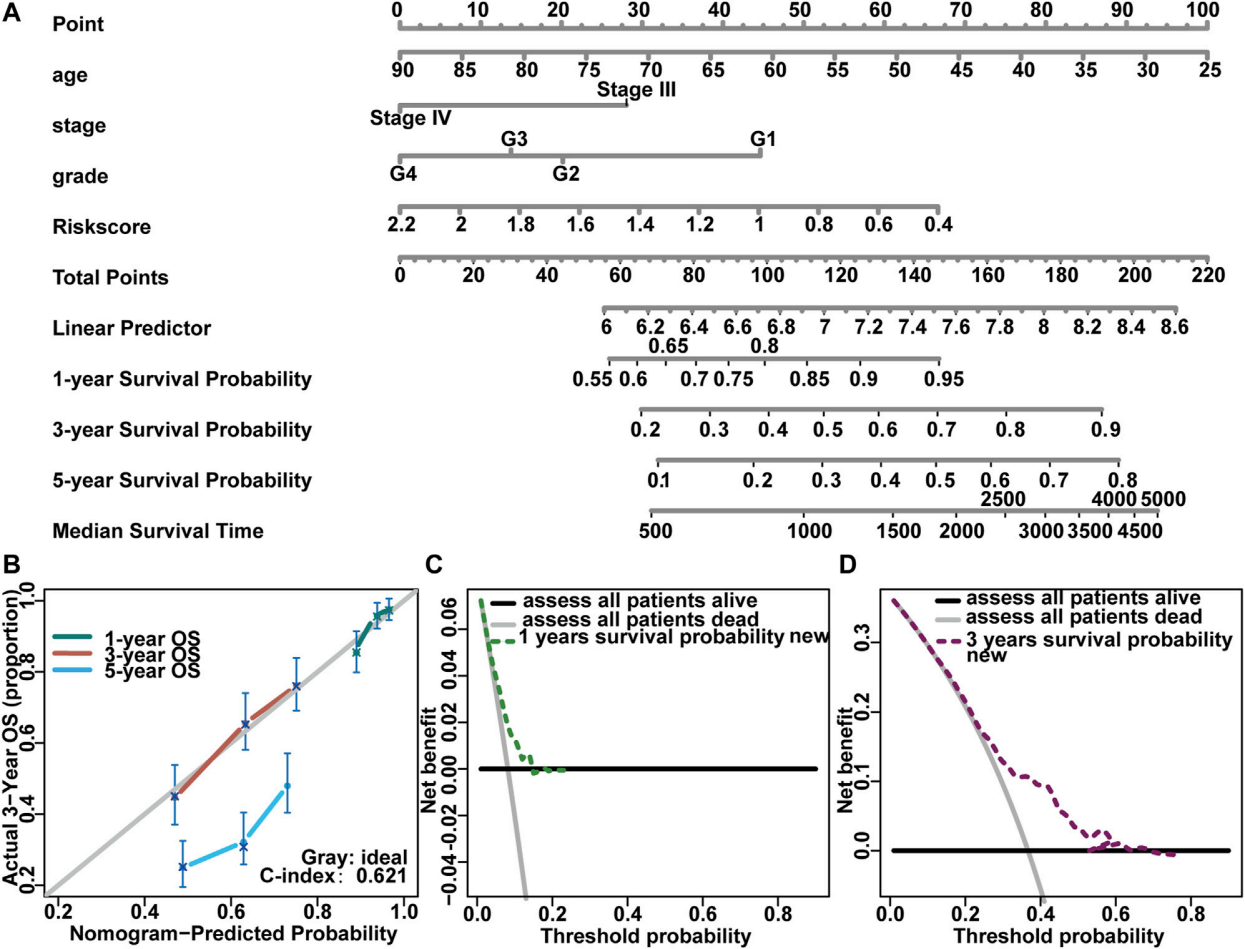
Construction of prognostic risk model and nomogram for genes with prognostic value. **(A)** The nomogram predicted overall survival in stage III-IV OV patients; **(B)** The calibration curves of nomogram evaluated OS showing the outcome of 1 year, 3 years, and 5 years; *x*-axis represented the nomogram prediction survival possibility, and *y*-axis represented the actual survival possibility; **(C, D)** Decision Curve Analysis evaluated the clinical utility of the nomogram. The *x*-axis represented the threshold possibility, and the *y*-axis represented the net benef.

We developed a model to assess survival in advanced ovarian cancer based on the risk score. We considered some clinicopathological factors such as age, stage, and grade in advanced ovarian cancer patients (stage III–IV) (Figure 10A). Then, we verified this result through the Concordance index (C-Index), Calibration plot (Figure 10B), and Decision Curve Analysis (DCA) (Figures 10C,D). The C-index in 1 year, 3 years, and 5 years were all 0.632. Corrected lines in 1 year and 3 years were close to the ideal curve, but the deviation was larger in 5 years. Further, the DCA was used to verify the 1 year and 3 years results. The 1 year survival curve was very close to the two extreme curves, and the 3 years survival curve was far from the two extreme curves.

## Discussion

As a potential treatment option, tumor immunotherapy has made great progress in recent years (Xu et al., 2021). Due to the molecular heterogeneity of serous ovarian cancer, the clinical prognosis of patients with the same stage may be very different. Prognostic evaluation based on immune characteristics is essential for appropriate treatment decisions. In this study, we screened the immune-related genes of serous ovarian cancer from a different perspective and discovered the importance of TAP1 and CXCL13 as independent prognostic and predictive markers for serous ovarian cancer. Moreover, the expression of TAP1 and CXCL13 were indeed increased in ovarian cancer tissues through experimental verification. Further, we have constructed an immune-related risk model based on TAP1 and CXCL13 for the first time to predict the prognostic response of advanced serous ovarian cancer.

Overall, four gene expression subtypes of serous ovarian cancers, which comprised 308 cancer samples from TCGA database with an exact genotype were found to have different immune and stromal scores. Mao et al. reported that the stromal score was related to the tumor microenvironment and it was an essential factor for the prognosis of gastric cancer (Mao et al., 2020). Moreover, Santoiemma et al. reported that the accumulation of tumor-infiltrating lymphocytes (TILs) in ovarian cancer can inhibit tumor progression, whereas high numbers of immunosuppressive regulatory T cells are associated with poor prognosis (Santoiemma and Powell, 2015). In the present study, the immunoreactive subtypes showed significant differences in OS when compared with the other three groups (immunoreactive vs. proliferative, *p* = 0.0264; immunoreactive vs. mesenchymal, *p* = 0.0009; immunoreactive vs. differentiated, *p* = 0.0123), which agrees with the results reported by an Australian study (Verhaak et al., 2013). The prognosis of the immunoreactive type was better (Konecny et al., 2014) primarily because of the infiltration of the tumor tissue by lymphocytes, whereas the mesenchymal type was characterized by more stromal hyperplasia and was associated with a poorer prognosis (Verhaak et al., 2013). Shilpi et al. also described survival differences between these four gene expression subtypes in the GEO database (overall *p*-value < 0.001) (Shilpi et al., 2019).

Next, based on the superiority of immune score and survival, gene expression differences were assessed via GO and KEGG functional enrichment analysis between the immunoreactive and other types. Overall, these differential genes were primarily involved in immune response and stromal components according to GO analysis, and the KEGG pathway also revealed that they were primarily involved in infection, inflammation, and cancer, further confirming their close relationship with the tumor microenvironment. Di et al. reported that the DEGs between high and low immune-score groups in glioblastoma were primarily contributing to immune response and matrix formation, which were the essential factors in the tumor microenvironment (Jia et al., 2018). Upon further analysis of 16 public cohort datasets (Hao et al., 2018), ovarian cancer with low immune scores were found to usually lacked chemokine and interferon-γ pathway genes. Moreover, a high immune score of ovarian cancer was substantially associated with *BRAC1/2* mutation status and outstanding response to immunotherapy (Hao et al., 2018). Yoshihara et al. also revealed that the level of immune activation genes was positively related with the overall survival of high-grade serous ovarian cancer (Yoshihara et al., 2012).

Herein, DEGs shared by the three OV groups were revealed *via* a Venn diagram, among which 14 genes were found to be associated with better prognosis in ovarian cancers by univariate survival analysis (HR < 1, *p*-value < 0.05). These genes with prognostic significance mainly focused on immune response, inflammatory response, and protein metabolism. Protein-protein interaction analysis showed that chemokine family members CXCL9, CXCL11, and CXCL13, which are important mediators of leukocyte migration to inflammatory sites (Palomino and Marti, 2015), formed the only MCODE component. Long-term inflammatory responses, such as chemoattraction to T cells and NK cells, as well as the recruitment of B cells to tumors are essential for immune infiltration and the formation of tertiary lymphoid structures that can provide an ideal microenvironment for antitumor immunity (Tian et al., 2017; Hao et al., 2018; Kazanietz et al., 2019; Kerdidani et al., 2019; Workel et al., 2019).

Finally, all 14 genes identified herein were found to have prognostic value, as confirmed by internal validation. Using an independent GEO cohort of 260 high-grade serous ovarian cancer samples, 12 genes showed significant correlations between gene expression and overall survival. TIMER showed that these validated genes were positively correlated with TILs and inversely proportional to tumor purity. Similar results were reported by Rhee et al. (2018). To date, immune marker genes have been emphasized as potential targets for immunotherapy, with accumulation of TILs being a positive survival prognostic indicator in ovarian cancer (Tsiatas et al., 2009; Hamanishi et al., 2011; Le Page et al., 2012; Li et al., 2017b). A meta-analysis comprising 2,903 ovarian cancer patients showed that intraepithelial CD3^+^ and CD8^+^ TILs are closely related to improved PFS, disease-free survival, and OS (Li et al., 2017b). Other reports showed CD4^+^ TILs also have prognostic value in ovarian cancer (Tsiatas et al., 2009; Hamanishi et al., 2011; Le Page et al., 2012). Moreover, a study of 199 ovarian cancer patients showed that the presence of CD20 ^+^ B cells could improve patient survival (Milne et al., 2009), as B cells can promote the production of cytokines to induce the local lymphoid structure of the tumor, thereby stimulating the persistence of CD8^+^ TILs that will promote tumor cell lysis (Nielsen and Nelson, 2012).

Among the herein validated genes, TAP1 and CXCL13 were identified as positive risk factors through the Cox proportional-hazards model. TAP1 encodes a transporter that is responsible for presenting tumor antigens in major histocompatibility complex I or human leukocyte antigen complexes (Tabassum et al., 2021). Hence, TAP1 mutation may be used as a target for immunotherapy in cancer patients (Tabassum et al., 2021). Anka et al. showed that TAP1 was overexpressed in breast, lung, and ovarian cancer, with the frequency of TAP1 alterations in ovarian cancer being the highest among the four types of cancers (Tabassum et al., 2021). CXCL13 is a chemokine ligand produced by follicular dendritic cells (Li et al., 2020). Fan et al. analyzed the correlation between CXCL13 and the immune invasion and TMB in ovarian cancer, revealing that CXCL13 was related to high TMB (Fan et al., 2020b). To date, some studies have shown the significance of TAP1 or CXCL13 as reliable immune-related prognostic genes in ovarian cancer (Liu et al., 2020; Wu et al., 2020; Huo et al., 2021; Li et al., 2021). Li et al. found that CXCL13 combined with CCL18, HLA-DOB, HLA-DPB2, and TNFRSF17 is associated with better overall survival in ovarian cancer between high and low abundance immune subtypes (Li et al., 2021). Huo et al. identified 10 immune microenvironment genes including TAP1 related to the prognosis of ovarian cancers (Huo et al., 2021). Although the positive correlation between TAP1 and CXCL13 was weak in ovarian cancers according to the cBioPortal database, some studies still screened that they can be both used as prognostic markers of ovarian cancer (Liu et al., 2020; Wu et al., 2020). Different from the univariate Cox analysis (Wu et al., 2020), Liu et al. eliminated the interference of other genes and 10 OS-related prognostic genes were extracted from the DEGs between ovarian cancer and normal tissues (Liu et al., 2020). In this study, we try to use a more concise way to predict the OS of ovarian cancers. We have found two immune-related genes, just TAP1 and CXCL13, which can be used as independent prognostic factors for ovarian cancer.

As the frequently altered neighbor genes of TAP1 and CXCL13, the immune-related genes CTLA-4, IFNG, and PRF1 were found to have the higher interconnected nodes according to the protein-protein interaction network. CTLA-4 has served as targets of immunotherapy by immune checkpoint pathways (Yap et al., 2021), whereas IFNG and PRF1 are both cytotoxic genes positively correlated with CXCL11 expression (Cao et al., 2021). Interestingly, IFNG was also reported to have a critical role in chimeric antigen receptor T cell-mediated endogenous immunity induction (Alizadeh et al., 2021). As an important prognostic factor, PRF1 was associated with immune infiltration in head and neck cancer (Fan et al., 2021).

Herein, the Tumor-Node-Metastasis staging system was used to evaluate the survival outcome of patients; however, patients with the same anatomical distribution were classified into the same stage without considering other variables, such as genetic differences or histology (Balachandran et al., 2015). Nomograms are widely used as a prognostic tool in oncology (Cho et al., 2008). One of the main advantages of nomograms is that it can help estimates individualized risks based on specific characteristics of patients and the disease, thereby aiding clinical decisions from different perspectives (Kattan et al., 2002; Wang et al., 2008; Gold et al., 2009; Zivanovic et al., 2012). Based on risk factors and clinically relevant characteristics, a nomogram model was established using a large number of TCGA samples to determine whether additional treatment was needed. First, the calibration curve evaluated the prediction probability. Overall, the predicted probabilities of 1- and 3 years were consistent with current reports, but the 5 years survival rate was lower than the actual value. Second, the concordance index was further used to evaluate the prediction accuracy. The concordance index is 0.638, illustrating the model has some accuracy. Third, DCA was the simple method used herein to assess clinical prediction models when considering the clinical utility of specific models (Vickers and Elkin, 2006). The 1 year Nomogram was found to have a narrower threshold probability with less clinical utility, whereas the 3 years nomogram may have clinical utility.

In general, this study highlights the differences in immune scores and survival prognosis between the immunoreactive and other gene expression types in serous ovarian cancers. In particular, the common differential genes with prognostic significance were determined, and their correlation with immune infiltrating cells was described. Finally, a nomogram model for advanced ovarian cancer based on TAP1 and CXCL13 was established for the first time. Although the accuracy of this nomogram model was verified. Its predictive effect still needs to be further investigated in the basic experiment and large-scale multicenter clinical trials.

## Data Availability

The original contributions presented in the study are included in the article/Supplementary Material, further inquiries can be directed to the corresponding author.
