# Supplementary material for: Gene Expression Subtyping Reveals Immune alterations:TCGA Database for Prognosis in Ovarian Serous Cystadenocarcinoma
Source: Front Mol Biosci. 2021 Sep 24;8:619027. doi: 10.3389/fmolb.2021.619027 (PMC8497788; doi:10.3389/fmolb.2021.619027)
Supplement: Supplementary file 1 [file Table1.docx]

**Supplementary Material**

| **Supplementary Table S1. Clinical characteristics of the OV patients in TCGA and GSE32062** | | |
| --- | --- | --- |
| Characteristic | TCGA | GSE32062 |
| age , No. (%) |  |  |
| <58.5 | 272 (50%) |  |
| >58.5 | 276 (50%) |  |
| stage , No. (%) |  |  |
| I | 14 (3%) | 0 |
| II | 26 (5%) | 0 |
| III | 426 (78%) | 204 (78%) |
| IV | 82 (15%) | 56 (22%) |
| grade , No. (%) |  |  |
| G1 | 6 (1%) | 0 |
| G2 | 69 (13%) | 131 (50%) |
| G3 | 462 (84%) | 129 (50%) |
| G4 | 1 (0%) | 0 |
| GX | 10 (2%) | 0 |
| OS , No. (%) |  |  |
| dead | 329 (60%) | 121 (47%) |
| live | 219 (40%) | 139 (53%) |
